# Association analysis between *HOXD9* genes and the development of developmental dysplasia of the hip in Chinese female Han population

**DOI:** 10.1186/1471-2474-13-59

**Published:** 2012-04-20

**Authors:** Wei Tian, Lixi Zhao, Jing Wang, Peisu Suo, Jianmin Wang, Longfei Cheng, Zhi Cheng, Jian Jia, Shilian Kan, Binbin Wang, Xu Ma

**Affiliations:** 1 Tianjing medical University, Tianjing, China; 2 Department of Orthopaedic Trauma, Tianjin hospital, Tianjin, China; 3 Graduate school, Peking Union Medical College, Beijing, China; 4 Center for Genetics, National Research Institute for Family Planning, 12, Dahuisi Road, Haidian, Beijing, 100081, China; 5 Huabei oil field hospital, Hebei, China; 6 World Health Organization Collaborating Centre for Research in Human Reproduction, Beijing, China

**Keywords:** Developmental dysplasia of the hip (DDH), HOXD9, Tag SNP, Association analysis

## Abstract

**Background:**

Developmental dysplasia of the hip (DDH) is a congenital or acquired deformation or misalignment of the hip joint which affects mainly females. We hypothesized that *HOXD9* gene could be regulated in acetabular size or shape and related in DDH developing.

**Methods:**

Two hundred and nine Chinese Han female DDH patients and 173 ethnic, age matched healthy female controls were genotyped for *HOXD9* two tag SNPs using sequenom method.

**Results:**

One of the two tag SNPs, rs711822, was not shown significantly differences in genotypic or allelic distribution between case and control group. Comparing the genotypic distribution of rs711819, there was significant differences between DDH patients group and control group (*χ*2 = 7.54, df =2, *P* =0.023), and the association to DDH developing reached significance (*P* =0.045, OR =1.79, 95 % CI: 1.01-3.17 by dominant mode).

**Conclusion:**

In conclusion, the association between one tag SNP of *HOXD9* gene and the development of DDH reach significant in our study population, this result indicate the positive correlation between *HOXD9* gene and DDH developing. Further study in larger sample size and different population as well as functional studies will help to understand the pathogenesis of DDH.

## Background

Developmental dysplasia of the hip (DDH) or congenital dislocation of the hip (CDH) (MIM number: 142700) is a congenital or acquired deformation or misalignment of the hip joint; include subluxation, dysplasia and dislocation [1] which affect approximately between 1 and 5 per 1000 in China [2]. DDH is an abnormality of the seating of the femoral head in the acetabulum [3], affects mainly females (sex-ratio: 8:1), and is a common causing of childhood disability or adult secondary hip osteoarthritis (OA) gradually [4,5].

Breech presentation (especially vaginal delivery), primiparity and high birthweight have been identified as related factors of DDH developing [6]. Apart from this, family studies have provided evidence that there is a genetic predisposition to DDH based on polygenic-multifactorial inheritance [7].

The homeo box (Hox) gene families encode highly conserved transcription factors which have been implicated in a variety of patterning events during vertebrate skeleton development. Altogether 39 Hox genes have been identified in mammalian genome and were clustered in four genomic loci (HoxA-D) [8]. Hox proteins bind specific DNA sequences via the homeodomain, and are thought to regulate overlapping sets of target genes, although the molecular pathways controlled by Hox proteins are only starting to be unveiled [9].

The homeo box D (HOXD) genes form a cluster at 2q24.1-q33.1, which contain 9 genes (HOXD1, 3, 4, 8–13). HOXD gene cluster is involved in skeletal morphogenesis, especially *5′ HOXD* gene (paralogous groups 9–13) play a crucial role in limb development [10]. Targeted mutagenesis and misexpression of individual and multiple *HoxD* genes in the animal model (mouse and chick) showed that the perturbation of Hox gene expression alters instead the size, shape of specific skeletal elements [11,12].

The expression of *HOX* genes are spatial and temporal co-linearity. Combinations of Hox proteins are thought to specify individual segments of the appendicular skeleton. In the hind limb, *HOX10* paralogous genes are required to pattern the stylopod, and *HOX11* paralogous genes are required to pattern the zeugopod [13]. The hip joint is located in the proximal limb, suggesting that the distribution area of the 9^th^ pairs of HOX gene expression may be related to hip region coincide.

Deduced from the reason mentioned above, we hypothesized that *HOXD9* gene could be regulated in acetabular size or shape and related in DDH developing.

In order to determine whether *HOXD9* gene may play a role in DDH, we set up a genetic-based association study between *HOXD9* gene tag SNPs (single nucleotide polymorphism) simultaneously and DDH development in Chinese Han female population.

## Material and methods

### Subjects

Unrelated DDH female patients (n = 209, age (month, Mean ± SD) = 5.81 ± 3.55) were mainly selected by surgeons from several hospitals in Tianjin Hospital. All patients were sporadic cases and there weren't family data. All patients had been diagnosed as having DDH (unilateral: 198 or bilateral: 11), which was confirmed on the basis of clinical criteria in addition to ultrasound and radiographic evidence. Cases with systemic syndrome were excluded from the study. Unrelated age, healthy, ethnically matched female children (n = 173, age (month, Mean ± SD) = 6.47 ± 4.15) were included as controls. Informed consent was obtained from patients' parents or guardians. The study protocol conforms to the ethical guidelines of the 1975 Declaration of Helsinki and was approved by the Ethics Committee of the National Research Institute for Family Planning.

### Laboratory methods and selection of SNPs

Blood samples from patients and healthy controls were collected and stored at −20°C. Genomic DNA was extracted from peripheral blood leukocytes according to standard methods.

Following the data release from Phase II of the International HapMap project [14], the selection of tag SNPs based on publicly available genotypes became a cost-effective option. Sample based genotypes (data release 24) were downloaded for all variants in genetic and 2000 bp promoter regions surrounding *HOXD9* gene.

Since the study populations under investigation were Chinese population, downloaded genotypes were restricted to those for the CHB (Han Chinese in Beijing, China) population. Tag SNPs were selected using a pairwise tagging algorithm and r^2^ exceeded 0.8 for all downloaded SNPs with minor allele frequency (MAF) > 5 % [15]. PCR primers were designed using iPLEX GOLD (Sequenom MassARRAY®). The primers of SNPs for mass spectrometry are showed as follow: rs78466921 (1st-PCR Primer CATTCGGCGGTTTTGA; 2nd-PCR Primer CATTCGGCGGTTTTGT), rs6745764 (1st-PCR Primer GGGGAACGACAGCACAC; 2nd-PCR Primer GGGGAACGACAGCACAT), rs35817516 (1st-PCR Primer aTCGGGCAGTGAAGGTC; 2nd-PCR Primer aTCGGGCAGTGAAGGTT), rs7601234 (1st-PCR Primer CAGCGACATCTACTACGC; 2nd-PCR Primer CAGCGACATCTACTACGA), rs711812 (1st-PCR Primer CCAGCAGAGCCAAATCAG; 2nd-PCR Primer CCAGCAGAGCCAAATCAT), rs711815 (1st-PCR Primer AAGCTCATGGAACGATCAC; 2nd-PCR Primer AAGCTCATGGAACGATCAT), rs4972803 (1st-PCR Primer gTCTAAGGGAGGATGACTA; 2nd-PCR Primer gTCTAAGGGAGGATGACTG), rs847153 (1st-PCR Primer cTCCCAGTTCCTAGATACTC; 2nd-PCR Prime cTCCCAGTTCCTAGATACTT), rs711822 (1st-PCR Primer AGCGACTTGGACTTCTTCTTC; 2nd-PCR Primer AGCGACTTGGACTTCTTCTTT), rs847194 (1st-PCR Primer TCAGGTTTCTTAGGAAAAAGG; 2nd-PCR Primer TCAGGTTTCTTAGGAAAAAGT), and rs711819 (1st-PCR Primer AGCTAACAGAAAATTCGTTACCC; 2nd-PCR Primer AGCTAACAGAAAATTCGTTACCT). Matrix-assisted laser desorption/ionization Time-of-flight Mass Spectrometry (MALDI-TOF MS) was used to perform multiple PCR and using SAP to digest the remanent dNTP in the system to avoid the affecting of single base extension. The PCR products after single base extension were purified and using MALDI-TOF MS to perform genotypic analysis.

### Genetic and statistical analysis

Allele frequencies and genotype distribution of patients and controls were determined by allele-counting method and analyzed with Pearson chi-square tests using Statistic Package for the Social Science (SPSS) version 10.0. To assess the strength of relationship in the allele distribution, the odds ratios (OR) and 95 % confidence intervals (95 % CI) were computed. First, we analyzed the Hardy-Weinberg equilibrium of SNP rs711822 and rs711819 in both patient group and control group. Then, the allele frequencies and genotype distribution of the two tag SNPs were compared between patients and controls. Finally, the haplotype frequencies of the two tag SNPs were calculated and analyzed. And we defined the LD map of CHB population in Hapmap databases as the reference category. The estimated statistical significance was *P* < 0.05.

## Results

The participants of the present study were matched with age. Two SNPs were selected using above-mentioned methods to pick tag SNPs, rs711822 was a intronic SNP while rs711819 was located at the promoter region of *HOXD9*. Both of them could be designed in a single well for genotyping.

According to the result (illustrated in table 1), the distributions of allele frequencies of two involved SNPs, were in Hardy-Weinberg equilibrium in both patient group and control group. There were no significant differences between the distributions of allele or genotype frequencies of rs711822 which located at the intronic region of *HOXD9* gene. Comparing the genotypic distribution of rs711819, there was significant differences between DDH patients group and control group (*χ*2 = 7.54, df =2, *P* = 0.023). Furthermore, the association to DDH developing reached significance (*P* = 0.045, OR =1.79, 95% CI: 1.01-3.17 by dominant mode).

**Table 1:** Genotype distribution, relative allele frequencies and haplotype data of HOXD9 tag SNPs

Single Marker		Allele					Genotype				
rs711822		A (freq)		G (freq)		A/A (freq)		A/G (freq)		G/G (freq)	
	case	164 (0.392)		254 (0.608)		27 (0.129)		110 (0.526)		72 (0.344)	
		unilateral	bilateral	unilateral	bilateral	unilateral	bilateral	unilateral	bilateral	unilateral	bilateral
		152(0.384)	12(0.545)	244(0.616)	10(0.455)	24(0.121)	3(0.273)	104(0.525)	6(0.545)	70(0.354)	2(0.182)
	control	131 (0.379)		215 (0.621)		27 (0.156)		77 (0.445)		69 (0.399)	
		*X*^2^ = 0.15, df = 1, *P* = 0.697					*X*^2^= 2.52, df = 2, *P* = 0.284				
rs711822		A (freq)		G (freq)		A/A (freq)		A/G (freq)		G/G (freq)	
	case	258 (0.617)		160 (0.383)		73 (0.349)		112 (0.536)		24 (0.115)	
		unilateral	bilateral	unilateral	bilateral	unilateral	bilateral	unilateral	bilateral	unilateral	bilateral
		246(0.621)	12(0.545)	150(0.378)	10(0.455)	70(0.354)	3(0.273)	106(0.535)	6(0.545)	22(0.111)	2(0.182)
	control	207 (0.609)		133 (0.391)		69 (0.406)		69 (0.406)		32 (0.188)	
		*X*^2^ = 0.056, df = 1, *P* = 0.813				*X*^2^= 7.54, df = 2, **P** = 0.023							
*P* = 0.045, OR = 1.79,95 % CI: 1.01-3.17 by dominant mode													
Haplotype		G-A		A-G		G-G		A-A					
rs711822-rs711819	Freq	0.595		0.366		0.02		0.019					
		case	control	case	control	case	control	case	control				
		0.584	0.607	0.358	0.376	0.025	0.015	0.032	0				
	P value	0.5533		0.6454		0.3716		0.0045					

The same LD map was detected in control group when comparing with CHB population in Hapmap database (r^2^ =0.842). Considering the two tag SNPs together, though the *P* value of the A-A haplotype was 0.0045, however the A-A haplotype frequency in control group was 0.

## Discussion

The etiology of DDH disease remains unknown, despite its prevalence. DDH is a complex disorder with both environmental and genetic causes. The primary finding of the present study, we establish the association between one tag SNP located at the promoter region of *HOXD9* gene and the development of DDH in Chinese female population. But it cannot be identified that the A-A haplotype was significantly associated with the presence of DDH because of the extremely small and unstable set of data. Thus, the studies of large sample size and different populations as well as functional experiments were needed to confirm the association between *HOXD9* gene and DDH developing. So far, we had a preliminary result to regard *HOXD9* gene may be a candidate gene for DDH developing.

Hypothetically, genes or genomic polymorphisms which affect acetabular morphology and capsular laxity potentially relate to the development of DDH.

The function of *Hox* genes at a later phase of limb development is less well defined but is likely to involve the regulation of cell differentiation. *Hox* genes suppress chondrogenesis directly and indirectly in the limb bud [16,17], which indicate that *Hox* genes not only have a role in early patterning processes but are also important for bone cell differentiation.Thus, *Hox* genes have an instructive role in determining the shape and ossification patterns of bones.

With regard to *HOX9* paralogous genes and DDH developing, *HOXB9* gene has been reported with contradictory results. In 2003, Jiang et al. firstly identified that *HOXB9* gene may be a susceptibility gene of DDH [18]. Rouault et al. reported in 2009 that there is no relationship between *HOXB9* and DDH [19]. Recently, a genome-wide screening of one 18-member, multigeneration family with DDH identified a linkage on a limited location of the specific chromosome 17q21 where *HOXB9* gene located in [20]. Besides, there has been no report on *HOXD9* to date.

In chicken model, the earliest Abd-B-like *Hoxd* gene expression bserved during limb bud outgrowth is the uniform activation of *Hoxd9* and *Hoxd10* along the entire anterior/posterior extent of the early limb bud [11]. Where after, *Hoxd11Hoxd12* and *Hoxd13* are activated sequentially at the posterior border of the limb bud [11].

In mouse, the importance of *HoxD* genes for normal skeleton growth and patterning has been established through loss and gain of function experiments [16]. Meanwhile, mutational analysis of some of these genes has demonstrated that they play an important role in limb development [21].

According to the result of the present study, we speculated the mechanism between the relationship of *HOXD9* gene and the development of DDH as follows:

Firstly, *HOXD9* gene regulate muscle cell growth, proliferation, differentiation and innervation, it can be speculated that these gene abnormalities cause muscle denervation, resulting in muscular dystrophy, or muscle fiber type and quantity of muscle leads to the malformation.

Secondly, *HOXD9* gene possibly induced the undifferentiated mesenchymal cells differentiate into cartilage and new bone via *BMP* gene family. Thus, this gene can affect the acetabular shape, the ossification groove development and the positioning of the femoral head.

## Conclusion

In conclusion, we established the association between tag SNP of *HOXD9* gene and DDH developing in a Chinese female Han population. Furthermore, these results indicate that this gene may be a new candidate gene of DDH disease. Follow-up studies involving deep investigating the loci as well as functional experiments will be needed to unravel the exact mechanism by which this region is pathologically involved.

## Abbreviations

DDH: developmental dysplasia of the hip; SNP: single nucleotide polymorphism.

## Misc

Wei Tian, Lixi Zhao, and Jing Wang contributed equally to the work.

## Competing interests

The authors declare that they have no competing interests.

## Authors' contributions

Jing Wang: data analysis, manuscript writing, experiment performing

Wei Tian: clinical consulting, sample collection

Peisu Suo: experiment performing

Jianmin Wang: sample collection

Longfei Cheng: experiment performing

Zhi Cheng: revised the manuscript

Jian Jia: revised the manuscript

Shilian Kan: sample collection

Binbin Wang: project design

Xu Ma: project design

Lixi Zhao: experiment performing

## Pre-publication history

The pre-publication history for this paper can be accessed here:

http://www.biomedcentral.com/1471-2474/13/59/prepub
